# Acute Kidney Failure and Hemolysis Secondary to High‐Dose *Teucreum polium* (Lamiaceae): Case Report

**DOI:** 10.1002/ccr3.70294

**Published:** 2025-03-02

**Authors:** Leila Gholami, Seyed Majid Ahmadi, Sheida Pursusan

**Affiliations:** ^1^ School of Nursing Yasuj University of Medical Sciences Yasuj Iran; ^2^ Department of Internal Medicine, School of Medicine Yasuj University of Medical Sciences Yasuj Iran; ^3^ School of Medicine Yasuj University of Medical Sciences Yasuj Iran

**Keywords:** acute kidney failure, case report, hemolysis secondary, *Teucreum polium*

## Abstract

Traditional medicine is becoming more popular worldwide. *Teucrium polium* belongs to the mint family and is a medicinal plant known for reducing blood sugar, hyperlipidemia, gastrointestinal tract issues, blood pressure, and urinary tract infections. High doses of this drug may cause liver and kidney toxicity, but this toxicity has not been thoroughly investigated. In this case report, we describe hemolysis and renal failure resulting from Arbitrary and traditional consumption of a high amount of *Teucreum polium* by the patient. After three cups of boiled *Teucreum polium* (each cup is approximately 200 cc) were administered to a 45‐year‐old white woman with an underlying hypertension disease with the intention of abortion, she developed abdominal pain, weakness, lethargy, nausea, fever, chills, anorexia, and hematuria. Laboratory investigations revealed severe hemolysis (LDH = 3903) and a decrease in hemoglobin to 4.7 mg/dL in 12 h. There was also a significant increase in the WBC, Retic count, BUN, and creatinine. A peripheral blood smear revealed +3 schistocytes and acanthosis. Viral markers, indirect coombs, and direct coombs were all negative. The primary diagnosis was acute intravascular hemolysis with renal failure. During treatment, with blood transfusion and hydration of the patient, liver and kidney function gradually improved. Due to the toxicity of medicinal plants, the unsupervised use of medicinal plants should be avoided.

**Summary**

- Although many medicinal plants have many benefits and some industrial drugs are extracted from these plants, in addition to imposing costs on patients, due to their harmful and unknown effects, these plants will lead to risks for consumers that should be considered and informed about this.

## 1 Introduction

Hemolytic anemia is caused by the decreased survival of circulating red blood cells (RBCs) due to premature destruction. Hemolysis can be intravascular or extravascular (such as in the spleen). The disease can be hereditary or acquired and can be mild to fatal. The main reason that hemolysis is the cause of anemia is an increase in the number of reticulocytes, which is not explained by recent bleeding or correction of iron or other nutrient deficiency or other causes (pregnancy, …). Patients may also have evidence of RBC destruction, including increased lactate dehydrogenase (LDH) and unconjugated bilirubin, decreased haptoglobin, and changes in RBC shape in peripheral blood smears [1, 2]. Medicinal plants have been used to treat various ailments since prehistoric times, and herbal medicine remains integral to many cultures worldwide. The side effects and resistance to currently used drugs have led to increased emphasis on using plant materials as a source of medicine for a wide range of human diseases [3], one of which is *Teucrium polium*.

*Teucreum polium* belongs to the mint family. This plant is traditionally used to treat various pathological conditions, such as digestive disorders, inflammation, diabetes, and rheumatism. It is also an antibacterial, antiulcer, blood pressure reducer, antispasmodic, antianorexia, and anti‐fever agent. Its therapeutic properties are due to the numerous chemical compounds in its essential oil [4, 5, 6]. *Teucreum polium* is used in traditional and herbal medicine worldwide [7, 8]; however, it has been found that this plant causes hepatotoxicity in humans [8, 9, 10]. Many studies have shown that the consumption of *Teucreum polium* causes toxicity and liver damage [11]. The mechanism of toxicity is related to the diterpenoids furano neochlorodan, tocrin A, and tecmodrin A, which have been reported as possible hepatotoxic precursors of this plant [12, 13]. One study showed that women should avoid consuming *Teucreum polium* plants during pregnancy because this plant can have very toxic effects in the early stages of pregnancy [14, 15]. In another report, liver damage has been associated with autoantibodies in the serum. Studies on the renal side effects of *Teucreum polium* are scarce [10]. A survey by Khelifat et al. showed that the continuous use of *Teucreum polium* for 6 weeks is associated with increased blood urea [16]. Rafieian‐Kopaei and Baradaran showed that injecting the aqueous extract of *Teucreum polium* by intraperitoneal injection results in toxic effects on the renal tubules: degeneration, destruction, and vacuolation. The presence of these metabolic byproducts tilts the balance in favor of oxidative stress, which exceeds natural antioxidants and increases the percentage of kidney damage either through nucleic acid alkylation or oxidation, protein damage, lipid superoxidation, and DNA strand breakage [17, 18].

## 2 Case History/Examination

The patient is a 45‐year‐old woman who came to the emergency department of Imam Sajjad Yasouj Hospital with complaints of abdominal pain, weakness and lethargy, headache, dizziness, nausea, anorexia, fever and chills, and hematuria. In the description of the present disease, the patient stated that due to a 4‐day delay in the menstrual cycle, with the suspicion of a possible pregnancy and for abortion, she consumed arbitrary and traditional consumption of 200 mL of *Teucrium polium* boiled in three cups. Three hours after consuming the *Teucrium polium* decoction, the patient experienced nausea, vomiting, abdominal pain, and hematuria. After the examination, the doctor noticed that the patient is yellow. According to the patient's symptoms, fluid therapy was started, and the patient was referred to Yasuj Medical Center for further investigation. However, the patient failed to perform the requested tests and returned home with a partial recovery of symptoms. Eight hours later, she returned to the hospital due to worsening symptoms, including weakness and lethargy, inability to walk, yellow eyes, and decreased urine output. After 12 h of taking *Teucrium polium*, the patient was admitted to Imam Sajjad Yasouj Hospital.

According to the patient's records, no points were related to anemia, allergies, G6PD deficiency, or surgery. The patient reported that she had been treated with 25 mg of Captopril daily for 5 years due to hypertension. Her menarche age was 14 years, her regular menstruation was 28 days, and her bleeding during each period was normal. The patient also mentioned a history of 5 deliveries. She also had a history of depression in previous years, in which she was treated with medicine, but she did not remember the name of the medicine. However, she mentioned that she had not taken any other medicine except Captopril in the past few years. The history of family diseases was also adverse.

## 3 Methods

The patient's vital signs included a blood pressure of 135.85 mmHg, a body temperature of 36.7°C, a heart rate of 77 beats/min, and an oxygen level of 91%–92%. In the examinations of the lungs, wheezing, galls, and decreased sounds were heard. In the study of the patient's abdomen, she mentioned tenderness in the left lower quadrant (LLQ) and epigastrium; the patient had no rebound; her abdomen was soft and did not express any history of diarrhea or constipation.

The results of the pregnancy test were negative in the initial tests. In the CBC diff test, a WBC of 21.9, ESR of 45, and CRP level of +2 were observed, which suggested the possibility of infection. However, there was no specific site of infection, and the patient was treated with meropenem. The inflammatory criteria favored infection, and broad‐spectrum antibiotics were started to treat the patient. Further, no evidence in favor of infection was found, and the patient did not mention a history of respiratory or urinary tract infection. In the tests, due to the decrease in hemoglobin (Hb = 4.7), it was suggested that the patient had anemia that was caused by poisoning and, along with the increase in MCV and Bili (Bili = 7.09, Direct Bili = 1.9 Total), LDH: 3667 and AST: 85, which suggested hemolysis in the patient, and a peripheral blood smear was prepared from the patient for further evaluation. RBC morphology in the patient's peripheral blood smear showed Aniso = +2, Hypochrom = +3, and fragmentation of 3%, which confirmed hemolysis in the patient. Viral markers, direct coombs, indirect coombs, and G6PD tests were performed to evaluate the patient further, and the results were negative. Coagulation tests in the patient were also standard. Concerning hemolysis, three units of blood were transfused into the patient on the sixth day of hospitalization. After the last pack cell transfusion, the patient's hemoglobin increased to 9.7 × 10^3^. (Table 1).

**TABLE 1:** Values of laboratory hematology tests of a patient with kidney damage and hemolysis caused by *Teucrium polium* during hospitalization.

Variable	The first day	The third day	The sixth day	The eighth day
WBC	21.9 × 10^3^	16.59 × 10^3^	9.7 × 10^3^	9.1 × 10^3^
RBC	1.42	2.60	2.83	2.59
HB	4.7	8.5	8.3	9
HCT	14.4	24.7	26	26.3
MCV	101.4	95	91.9	91.9
MCH	33.1	32.7	29.3	29.6
RDW	13.9	16.6	15.2	15
Retic count	4.2	1.6	2.3	1.9
ESR	45	—	—	—

Examination of kidney tests revealed a change in kidney function, which increased creatinine by 3.35 and urea (BUN) by 76.49, indicating acute kidney failure. Additionally, her kidney electrolytes were disturbed, so her sodium level was 131.2, but her potassium level was within the normal range (*k* = 3.7) (Table 2).

**TABLE 2:** Biochemical test values of a patient with kidney damage and hemolysis caused by *Teucrium polium* consumption.

Variable	First day of hospitalization	The fourth day of hospitalization	The sixth day of hospitalization	Eighth day (discharge)
BS	115	—	—	—
BUN	76.49	68.62	42.19	35
CR	3.35	5.59	4.42	1.8
NA	131.3	135.5	133.7	134
K	3.7	3.97	4.19	4.3
AST	85	16	15	16
ALT	30	13	10	10
Total protein	6.5	5.8	5.5	5.3
Total Bili	7.09	1.08	1.04	1.02
Direct Bili	1.9	0.23	0.18	0.16
LDH‐p	3667	1256	954	756
Amylase	100.7	—	—	—
Lipase	56.5	—	—	—

The acute kidney failure that developed in this patient may have been related to poisoning or cell lysis. To treat renal failure, the patient underwent hydration, and kidney function was monitored regularly. During hospitalization, after treatment, the BUN, Cr, and LDH levels started to decrease, and kidney function gradually improved. In the last evaluation of the patient 1 week after discharge, kidney function was expected, and liver enzymes and LDH were within the therapeutic range. CBC (Hb: 9.5, WBC: 9/6, plt: 180) BUN: 19, Cr: 1.2 indicated improved patient condition.

## 4 Discussion

*Teucreum polium* is used worldwide in traditional and herbal medicine. In some studies, kidney toxicity due to the consumption of *Teucreum polium* has been mentioned [8, 16]. However, for all known positive effects, the potential organ side effects, liver and kidney effects must be known for a ‘safe use’ of herbal or chemical drugs. Otherwise, herbal medicines, which are recommended for therapeutic purposes, may cause many unwanted side effects. Essential oils constitute 0.45% of *Teucreum polium*. It is also thought that the phenolic compounds in these essential oils have antidiabetic effects [19]. In this study, 2 days after taking *Teucreum polium*, the patient experienced acute intravascular hemolysis with renal failure and responded to treatment after 10 days. Given the uncertain effect of the plant on kidney function and the unknown damage to other organs, factors that exacerbate the patient's symptoms include failure to follow up with the patient and delay in referral. However, given that the primary mechanism of damage is unknown, this relationship cannot be proven with certainty, but it can also cause oxidative damage. Our patient was treated with blood transfusion and adequate hydration with intravenous fluids. Renal failure was probably secondary to intravascular hemolysis, which improved with supportive therapy.

## 5 Conclusion

Herbal and dietary supplements are popular worldwide, and herbal medicines are used not only in Asian countries but also in Europe and America; therefore, it is recommended that herbal medication be used with more caution. Although many medicinal plants have many benefits and some industrial drugs are extracted from these plants, in addition to imposing costs on patients due to their harmful and unknown effects, these plants will lead to risks for consumers that should be considered and informed about. Doctors should consider excellent care and sensitivity when dealing with these patients.

## Author Contributions

**Leila Gholami:** data curation, resources, writing – original draft, writing – review and editing. **Seyed Majid Ahmadi:** conceptualization, investigation, supervision. **Sheida Pursusan:** funding acquisition.

## Ethics Statement

This study was approved by the Research Ethics Committee of Yasouj University of Medical Sciences with code number IR YUMS.REC.1403.002.

## Consent

Written informed consent was obtained from the patient to publish this case report and any accompanying images.

## Conflicts of Interest

The authors declare no conflicts of interest.

## Data Availability

The authors have nothing to report.
